# Correspondence

**Published:** 1899-03

**Authors:** B. T. Whitmore

**Affiliations:** New York


### Correspondence.

New York, January 9, 1899.

The Editor Southern Medical Record, Atlanta, Ga.

Dear Sir: I note with interest your very brief remark of satisfaction with the past and hope for the future in beginning your twenty-ninth year. It is a circumstance on which you might properly have dwelt with greater insistence, for your old friends are thoroughly acquainted with what good work you have done in the literary field of medicine, and the new friends will soon be able from their own reading to reach the same conclusion. Let me congratulate you on the high position which the Record has attained under your direction and to express my sincere hope that it will win further approval. You will readily appreciate that I have a high regard for the success of efforts such as yours, for the medical periodical press stand in a confidential and educational relation to the many members of our profession whose circumstances of devotion to duty with often poor recompense prevent them from the advantage of systematic post-graduate instruction. To such medical men, and their number is great, the conscientiously edited medical journal is really a university.

I recall with pleasure Dr. Westmoreland, of your editorial staff, whose acquaintance I had the pleasure of making at the Atlanta meeting of the American Medical Association.

Very truly yours,

B. T. Whitmore, M.D., LL.D.

